# The Effectiveness of Virtual Training on the MiniMed™ 670G System in People with Type 1 Diabetes During the COVID-19 Pandemic

**DOI:** 10.1089/dia.2020.0234

**Published:** 2021-01-28

**Authors:** Robert A. Vigersky, Kevin Velado, Alex Zhong, Pratik Agrawal, Toni L. Cordero

**Affiliations:** ^1^ Medical Affairs, Medtronic Diabetes, Northridge, California, USA.; ^2^ Research and Development, Medtronic Diabetes, Northridge, California, USA.

**Keywords:** Diabetes, Diabetes technology, Diabetes education, Closed-loop systems, COVID-19, Virtual education

## Abstract

***Background:*** The coronavirus disease 2019 (COVID-19) pandemic has challenged the ability to do face-to-face training on advanced diabetes management technologies. In the United States, Medtronic Diabetes shifted from occasional to 100% virtual training on all diabetes devices in mid-March 2020. We studied the outcomes of virtual training on the MiniMed™ 670 G hybrid closed-loop system in type 1 diabetes.

***Methods:*** From March 20, 2020, to April 22, 2020 (intra-COVID-19), virtual training on the MiniMed 670 G system was completed using Zoom with satisfaction captured through online post-training surveys. Training efficiency was measuring by the days between the date of product shipment and the date of the first and final trainings. Patient satisfaction with training on the MiniMed 670 G was determined by Net Promotor Score^®^ (NPS^®^). Uploads from CareLink™ Personal and CareLink Professional and calls to the Medtronic 24-h technical support team requesting educational/software assistance and/or help with health care provider telehealth visits were recorded. Continuous glucose monitoring (CGM) results were measured using the CareLink Personal database. All results except for the Zoom satisfaction survey were compared with data from January 20, 2020, to February 22, 2020, (Pre-COVID-19) when training was performed in-person.

***Results:*** The CGM metrics were comparable between pre- and intra-COVID-19 training. The Zoom video conferencing application had 98% satisfaction. The NPS rose from 78 to 84. The time between the pump shipment and the first and last (automode) training was significantly reduced from 14 ± 7 days to 11 ± 5 days (*P* < 0.001) and from 19 ± 7 days to 15 ± 15 days (*P* < 0.01), respectively. There was a decrease in the calls for educational assistance to the technical support team but an increase in requests for login and software installation support.

***Conclusions:*** Virtual training of individuals with diabetes on the MiniMed 670 G system resulted in high satisfaction and short-term glycemic results comparable with in-person training.

## Introduction

The coronavirus disease 2019 (COVID-19) pandemic has upended the nonmedical and medical worlds in many ways. Overall, 39% of people in the United States have lost income and/or suffered layoffs in their household.^1^ For people with diabetes, there are numerous additional concerns ranging from the possibility of having an increased risk of contracting COVID-19 and the potential worsening of their prognosis to how they can optimize, if not adequately maintain, their current treatment. Although supply chains have remained intact,^2^ 55% of people with diabetes are concerned about obtaining their diabetes supplies.^3^ For those managing their diabetes with closed-loop systems, many of their health care providers (HCPs) expect that their patients would stop using continuous glucose monitoring (CGM) before stopping pump therapy if they lost their job.^4^

During this unprecedented time, HCPs face several challenges. Although 74% of endocrinologists continue to see patients, 20% of them are seeing only existing patients.^5^ Overall, there has been a 44% decline in patient visit volumes^5^ due to reluctance by many to have face-to-face encounters. In addition, many HCPs may not have access to their office notes, patient logbooks, and other tools such as fax machines that may be required to transmit prescriptions for advanced insulin management technology securely. In situations wherein prescription transmission is not an issue, some people with diabetes may have interrupted or have no access to insulin or consumables. Office staff and personnel who assist in preparing the information for pump therapy authorization may, also, not have access to the requisite clinical information.

Even in the event that insulin pumps and supplies are received, many device users have concerns about how they will learn to use their new therapy. Pump trainers and HCPs may question the feasibility of therapy training in a virtual environment, especially since training materials have not been specifically designed for remote training. In addition, the virtual one-on-one interface may not guarantee the undivided attention of the trainee at home, where there may be numerous distractions.

Medtronic Diabetes has provided virtual trainings on a case-by-case basis to a limited degree in the past. However, in mid-March 2020, all insulin delivery device trainings shifted to a virtual environment and Medtronic Diabetes has since trained thousands of individuals on insulin pumps with and without CGM devices. Herein, we report the results of the first 33 days of the virtual training approach on the MiniMed 670G™ hybrid closed-loop system from the standpoint of the patient trainee, HCP, and trainer and compare it with retrospective data before the COVID-19 era of sheltering-in-place and social distancing.

## Methods

This is a retrospective study of both clinical and process metrics comparing face-to-face training (January 20–February 22, 2020, i.e., pre-COVID-19) with virtual training (March 20 to April 22, 2020, i.e., intra-COVID-19). Glycemic outcomes data from CareLink™ Personal were collected on May 11, 2020, and on June 8, 2020, for all patients (with at least 10 days of SmartGuard™ auto mode usage) who were new to MiniMed™ 670 G system use during the pre-COVID-19 and the intra-COVID-19 eras. No IRB approval was sought.

The baseline therapy of the trainees was characterized as (1) MDI and SMBG; (2) MDI and sensor users; (3) pump and sensor users—upgrade of pump; and (4) pump and sensor users—upgrade of both. Training on the MiniMed 670 G system was conducted by Medtronic employees using the Zoom Enterprise Version of the Zoom video conferencing application (Zoom Video Communications, San Jose, California). The level of trainee and educator satisfaction with the application was captured using a postsession questionnaire. Medtronic employees delivered ∼96% of all training in the pre-COVID-19 era and 94% in the intra-COVID-19 era with the remainder of training accomplished by individual clinic staff. In-person training on the MiniMed 670 G system is usually done in three sessions of 90 min (pump), 60 min (CGM), and 60 min (SmartGuard technology) depending on the patient's current therapy and knowledge of diabetes management technology. The teaching materials during virtual training were the same as those used during the in-person training sessions.

We used two metrics as a proxy for the efficiency and effectiveness of the virtual training program. The first was the number of days between pump shipment and completion of the first training and the number of days between therapy start and the final training, and the second was the percentage of calls to the Medtronic 24-h technical support team that requested educational or software assistance and/or help with HCP telehealth visits. Those calculated from March 20, 2020, to April 22, 2020, (intra-COVID-19), were compared with those during pre-COVID in-person training days performed from January 20, 2020, to February 22, 2020, (pre-COVID-19).

We also compared the percentage of uploads to CareLink Personal versus CareLink Professional in the pre-COVID-19 era to the intra-COVID-19 era, as a proxy for the ability of HCPs and/or their staff to upload insulin pump data to professional accounts. The number of unique page views to the CareLink product page was measured during 2 weeks in the pre-Covid-19 era and compared with 2 weeks in the intra-COVID-19 period.

Patient experience was assessed with the Net Promoter^®^ Score (NPS^®^),^6^ a single-question management tool. The NPS is graded on a scale of −100 to +100 with values ≥50 regarded as excellent and ≥70 as “world class.” The two most relevant of the questions asked were (1) “based solely upon your recent training experience, how likely might you recommend Medtronic to another person who themselves are insulin-dependent (if they were considering pump therapy)”; and (2) “based upon all of your product and service experiences to-date, how likely might you recommend Medtronic to another person who themselves are insulin-dependent (if they were considering pump therapy)?”

Statistical analysis used the independent Student's *t*-test. Data are reported in percentages and/or percentage change rather than absolute numbers to protect trade secrets. However, the data are based on several thousand pump starts in each time period.

## Results

There were 6% of patients in the pre-COVID-19 era and 4% of patients in the intra-COVID era who did not receive training after the pump was shipped to them. The reasons were that the patients self-started, declined the training, or were unavailable and did not differ between time periods. The percentage of patients in each age range for the pre-COVID-19 and intra-COVID-19 time periods was 7–13 years—1% for both; 14–21 years—5% and 6%, respectively; and 22–80+ years—93% and 94%, respectively. Mean age for both cohorts was 47 years with 51% female and 49% male. The baseline therapy for each age group for the pre-COVID-19 and intra-COVID-19 by age is given in Table 1.

**Table 1.** Percentage of Patients Receiving Training Based on Prior Therapy by Age

		Prior therapy		
Age	MDI+sensor	MDI+SMBG	Pump upgrade	Pump and sensor upgrade
*Pre-COVID-19*				
7–13	22%	33%	28%	17%
14–21	8%	39%	16%	37%
22–80+	10%	23%	30%	37%
*Intra-COVID-19*				
7–13	0%	46%	23%	31%
14–21	19%	24%	27%	30%
22–80+	8%	14%	44%	34%

Pre-COVID-19 era is January 20, 2020, to February 20, 2020.

Intra-COVID-19 era is March 20, 2020, to April 22, 2020.

COVID-19, coronavirus disease 2019.

**FIG. 1.**
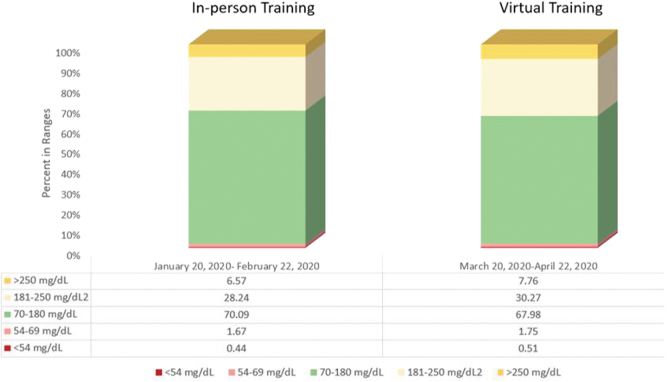
Times-in-ranges for people with diabetes newly trained on the MiniMed^TM^ 670 G system in-person in the pre-COVID-19 era (left) and virtually in the intra-COVID-19 era (right).

The glycemic outcomes for the pre-COVID-19 cohort and the intra-COVID-19 cohort are shown in Figure 1. Times-in-ranges (TIRs) data were obtained on May 11, 2020, which represented an overall time from final training to data collection of 3 and 1 months, respectively, and represent all data once the auto mode feature has been turned on for the first time. The data were retrieved again on June 11, 2020, to determine whether there was a difference with another month having elapsed (Table 2).

**Table 2.** Mean Sensor Glucose and Percentage of Time Spent in Sensor Glucose Ranges Overall and by Age Group

	All users		Age 7–13		Age 14–21		Age 22–49		Age 50–64		Age 65+	
		Pre-COVID users	Post-COVID users	Pre-COVID users	Post-COVID users	Pre-COVID users	Post-COVID users	Pre-COVID users	Post-COVID users	Pre-COVID users	Post-COVID users	Pre-COVID users	Post-COVID users
Percentage patients			1.6	1.8	4.9	8.8	50.6	53.1	36.1	27.4	6.6	8.9
Average SG	157.4 ± 15.3	160.2 ± 18.0	165.0 ± 25.0	174.2 ± 21.1	169.7 ± 23.8	172.1 ± 23.2	157.9 ± 15.1	160.6 ± 17.5	155.8 ± 12.9	157.0 ± 15.6	151.8 ± 10.9	152.9 ± 12.9
Percentage of time spent at ranges of SG, mg/dL												
<54	0.5 ± 0.8	0.5 ± 0.8	0.6 ± 0.5	0.6 ± 1.0	0.6 ± 1.8	0.8 ± 1.4	0.5 ± 0.8	0.5 ± 0.7	0.3 ± 0.5	0.4 ± 0.6	0.3 ± 0.5	0.4 ± 0.8
<70	1.7 ± 1.8	1.7 ± 1.8	2.3 ± 1.6	2.0 ± 2.1	1.8 ± 3.0	2.2 ± 2.6	1.9 ± 1.8	1.8 ± 1.7	1.4 ± 1.4	1.5 ± 1.5	1.3 ± 1.2	1.5 ± 1.7
70–180	70.4 ± 10.6	68.4 ± 11.9	63.60 ± 12.6	58.8 ± 11.4	62.7 ± 14.0	59.9 ± 13.0	69.5 ± 10.4	67.8 ± 11.5	72.1 ± 9.9	71.1 ± 10.9	75.0 ± 8.8	74.0 ± 10.3
>180	28.0 ± 10.4	29.9 ± 12.1	34.1 ± 13.6	39.2 ± 11.9	35.6 ± 14.1	37.9 ± 13.6	28.6 ± 10.5	30.4 ± 11.8	26.6 ± 9.8	27.4 ± 10.9	23.8 ± 8.7	24.5 ± 10.2
>250	6.5 ± 5.8	7.6 ± 7.0	11.7 ± 10.0	14.5 ± 8.6	12.3 ± 9.8	13.5 ± 10.0	6.7 ± 5.7	7.7 ± 6.6	5.5 ± 4.4	6.1 ± 5.8	4.2 ± 3.4	4.8 ± 4.6

SG, sensor glucose.

Overall, the glycemic results were similar between those getting in-person training compared with those receiving virtual training, although marginally better in the former cohort. There were minor but no clinically significant differences in the TIRs between the May and June data extractions. The TIR of 70–180 mg/dL increased progressively from the youngest to oldest group and the difference between the pre-COVID-19 and intra-COVID-19 era TIR narrowed from youngest to oldest. The time in auto mode was 94.9% and 95.5% in the pre-COVID-19 cohort and the intra-COVID-19 era, respectively. There were a slightly higher percentage of patients ≥65 years (8.9% vs. 6.6%) who received training in the intra- versus pre-COVID-19 eras (Table 2).

The number of days (mean ± standard deviation) between the pump shipment to patients (“start date”) and the first training (on the pump) was shorter in the intra-COVID-19 cohort than in the pre-COVID-19 cohort: 11 ± 5 days versus 14 ± 7 days (*P* < 0.001) (Table 3). The number of days between the pump shipment to patients (“start date”) and the third/final training (inclusive of the time to the first training) was 15 ± 15 days versus 19 ± 7 days (*P* < 0.01), respectively. The difference between the first and final training was 4 and 5 days in the pre-COVID-19 and intra-COVID-19 eras, respectively. There were no differences in the shortening of training completion (start date to third training) based on age (Supplementary Table S1).

**Table 3.** Duration of Time Between the MiniMed 670 G System Shipment Until the First and Final Training in the Pre-COVID-19 Era and Intra-COVID-19 Era

	In-person training (pre-COVID-19 cohort)	Virtual training (intra-COVID-19 cohort)
Date range	20 January–22 February	20 March–22 April
Mean time to first training, days (median; IQR)	14 ± 7 (13; 9–24)	11 ± 5^**^ (10; 7–17)
Mean time to final training, days (median; IQR)	19 ± 7 (20; 14–24)	15 ± 5^*^ (14; 11–17)

Pre-COVID-19 era is January 20, 2020, to February 20, 2020.

Intra-COVID-19 era is March 20, 2020, to April 22, 2020.

^*^ *P* < 0.01; ^**^P < 0.001.

The videoconferencing survey showed that the platform received a 98% satisfaction rating of “good.” The reasons for the 2% “not good” rating included poor video quality (41.1%), “they could not hear us” (31.3%), “we could not hear them” (8.9%), “we could not see them” (16.9%); and “other” (1.9%).

The NPS for question 1 was 78 in the pre-COVID-19 cohort (82.7% promoters, 12.3% passive, and 5% detractors) and 84 in the intra-COVID-19 cohort (87.6% promoters, 9% passive; 3.4% detractors). The NPS for question 2 was 74 in the pre-COVID-19 cohort (79% promoters, 15.9% passive, and 5.1% detractors) and 83 in the intra-COVID-19 cohort (86.2% promoters, 10.4% passive; 3.4% detractors).

In the first 2 months of 2020, ∼9% of calls to the 24-h technical support team were for educational and/or software support compared with 10.4% of calls between March 20, 2020, and April 22, 2020. The top five reasons for these calls were system feature inquiry, software assistance, and education on SmartGuard™ features and sensor glucose versus blood glucose differences. The details are shown in Supplementary Table S2. There was a 0–19% decrease in the percentage of calls for educational support in the intra-COVID-19 month (e.g., inquiries about system or sensor features and SmartGuard technology education). However, there was a 187% increase in software support inquiries ranging from installation to login assistance. There was a 92% increase in unique CareLink product page views observed, with >27,000 views occurring during 2 weeks in the intra-COVID-19 era compared with 2 weeks in the pre-COVID-19 era.

## Discussion

Recently imposed social distancing potentially impairs the ability of all stakeholders to initiate and utilize advanced insulin management technologies. However, since early March 2020, the data suggest that videoconferencing-enabled virtual training has been successfully employed by Medtronic employees. Operationally, the number of days from MiniMed 670 G system shipping to training completion has shortened, the subsequent follow-up calls for educational support to the technical support team have decreased, and patient satisfaction has increased.

Such outcomes were not intuitively obvious at the onset of this virtual journey. Delays in shipping due to limitations of package carriers, preoccupation of both patients with childcare, education, increase in time spent in virtual versus in-person work in those who were still employed, and schedule disruption may have made virtual training more difficult. Similar potential limitations apply to the trainers themselves. In hindsight, the success of the virtual training may be, in part, due to the use of a comprehensive curriculum and the availability of a 24-h technical support team but also related to patients not having to come to an office for training that may entail time away from their work or home obligations, fighting traffic, finding a parking space, and sitting in a waiting room. In addition, the favorable results may be due to the extra time that patients may have had to focus on the pump training tasks given their home sequestration.

Clinically, the glycemic metrics are only slightly lower in the intra-COVID-19 era with the use of the same curriculum in virtual training as is used in face-to-face training. One of the concerns of telehealth visits in general is that it may disadvantage those patients who are older because they are not as comfortable with technology. The data showing that the percentage of older patients who underwent virtual training was slightly higher in the intra- versus pre-COVID-19 era suggest that no substantive barriers to virtual training occurred in a potentially less technology savvy cohort although these patients may represent a higher overall comfort with technology given their desire to use a hybrid closed-loop system.

Although not directly related to the results of the switch from face-to-face to virtual training, use of CareLink has dramatically changed in the COVID-19 pandemic era. There was an overall 37% decrease in uploads to CareLink software in March–April compared with the same time period 1 year earlier. Eighty-one percent of this decrease was due to a reduction in uploads to CareLink Professional.

There was a 12.6% increase in uploads to CareLink Personal in the same time period. This is most likely due to fewer in-clinic visits, where HCPs and staff are relied upon to perform software uploads. This is mirrored by an increase in CareLink Personal use and a sharp increase in requests for software assistance and number of unique visits to the CareLink product page. This may be due to unfamiliarity with CareLink Personal software or because help was required with telemedicine needs unrelated to CareLink software. This also suggests that additional education may be needed to better prepare system users to perform regular uploads from home; an important practice even after the pandemic recedes.

The TIR and time-above-range (TAR) were marginally better in the pre-COVID-19 era cohort than in the intra-COVID-19 era cohort. This may be due to the greater number of days of use of the MiniMed 670 G system after face-to-face training, which may have afforded these patients more time to use the system and adjust their settings. We plan to re-evaluate data from these cohorts in 3 to 6 months that will allow the differences in the durations of time from training to CGM data collection on May 11 and June 7, 2020, to washout. There was also a high time in auto mode of 95% in both groups.

This is higher than real-world data of 87% as reported by Stone et al.^7^ The higher time in auto mode may reflect the newness of the therapy, enthusiasm for its use, and initial results as well as the correction of a recurrent alert called “BG loop” by introducing a new sensor transmitter in the past 2 years that eliminated this issue that forced recurrent exits from auto mode to calibrate the sensor with a blood glucose.

Another possible reason for these differences in TIR and TAR may be the fact that training materials were not changed for a virtual environment and that the time duration of each training session remained the same. Although multiple videos about the MiniMed 670 G system are available on the Medtronic website, there are plans to modify and optimize training materials for a virtual environment. It will also be important to assess the satisfaction level of prescribing HCPs with the virtual training. Supporting this speculation is the large increase in unique CareLink product page views.

In the post-COVID-19 world, there may be a new “normal” for how and where patients are treated. The telehealth market is expected to grow by 80% this year.^8^ This will likely include fewer in-person visits to clinics and more telemedicine visits, as long as payer reimbursement supports this switch. Since insulin pumps and CGM data can be uploaded from home and automatically accessed anywhere by an HCP, it is feasible for practically anyone to start a diabetes technology to manage their disease. This also affords an opportunity to help people living with diabetes in rural areas or endocrinology “deserts.”

There are other consequences of moving to a more virtual medical technology world. For example, it is likely there will be fewer device manufacturer representatives coming to clinics having been replaced by virtual HCP education and training sessions. The implications of job loss for device manufacturer employees and/or clinic employees are, as yet, to be determined. At the very least, there will be a change in the priorities and the requisite skills of medical professionals including improved competency in a wide range of telehealth technologies or platforms (e.g., Clarity^®^ software [Dexcom, Inc., San Diego, CA], t:connect^®^ Diabetes Management Application [Tandem^®^ Diabetes Care, Inc., San Diego, CA], Tidepool Uploader [Tidepool Project, San Francisco, CA], electronic medical record systems, and even DocuSign^®^ eSignature [DocuSign^®^, Inc., San Francisco, CA]).

The strengths of this study are the large data set used for analysis and the timeliness of the analysis. Limitations of the study include the inability to provide absolute numbers for the analysis, because they would reveal confidential business material or disclose proprietary information. However, as already noted, there were thousands of training sessions in each time frame that provide reassurance that the data are meaningful.

## Conclusions

The data demonstrate that satisfaction, training effectiveness, and early glycemic outcomes are comparable with and in some cases better than those observed in the pre-COVID era. Virtual training for new users of advanced diabetes management technology is a viable method for the post-COVID-19 world.

## Supplementary Material

Supplemental data

Supplemental data
