# Supplementary material for: The Effectiveness of Virtual Training on the MiniMed™ 670G System in People with Type 1 Diabetes During the COVID-19 Pandemic
Source: Diabetes Technol Ther. 2021 Jan 28;23(2):104–9. doi: 10.1089/dia.2020.0234 (PMC7868572; doi:10.1089/dia.2020.0234)
Supplement: Supplemental data [file Supp_Table1.pdf]

Supplementary Data

Supplementary Table S1. Duration of Time Between the MiniMed 670 G System Shipment Until the First and Final Training in the Pre-COVID-19 Era and Intra-COVID-19 Era, According to Age

	In-person training (pre-COVID-19 cohort)	*Virtual training (intra-COVID-19 cohort)*
7–13 years	21.5 days	17.0 days
14–21 years	18.5 days	16.0 days
22–80+ years	19.2 days	15.3 days

Pre-COVID-19 era = January 20, 2020, through February 20, 2020.

Intra-COVID-19 era = March 20, 2020, through April 22, 2020.

COVID-19, coronavirus disease 2019.
