# Supplementary material for: The Effectiveness of Virtual Training on the MiniMed™ 670G System in People with Type 1 Diabetes During the COVID-19 Pandemic
Source: Diabetes Technol Ther. 2021 Jan 28;23(2):104–9. doi: 10.1089/dia.2020.0234 (PMC7868572; doi:10.1089/dia.2020.0234)
Supplement: Supplemental data [file Supp_Table2.pdf]

Supplementary Table S2. Percentage of Pre-COVID-19 Era and Intra-COVID-19 Era Calls to the 24-H Technical Support Team for Educational Assistance or Support

	In-person training (pre-COVID-19 cohort)	*Virtual training (intra-COVID-19 cohort)*	*Change*
System feature inquiry	6.1%	5.3%	−13%
Software assistance[a]	1.5%	4.3%	+187%
Education			
SmartGuard™ technology	0.8%	0.6%	−19%
Sensor versus blood glucose	0.2%	0.2%	0%

[a]Includes software installation and explanations, as well as username or password factors.
Pre-COVID-19 era = January 20, 2020, through February 20, 2020.
Intra-COVID-19 era = March 20, 2020, through April 22, 2020.
